# Circ_0002984 induces proliferation, migration and inflammation response of VSMCs induced by ox‐LDL through miR‑326‐3p/VAMP3 axis in atherosclerosis

**DOI:** 10.1111/jcmm.16734

**Published:** 2021-06-25

**Authors:** Ruogu Li, Qiliang Jiang, Yue Zheng

**Affiliations:** ^1^ Department of Cardiovascular Surgery Shanghai Chest Hospital Shanghai Jiao Tong University Shanghai China; ^2^ Department of Anesthesiology Shanghai Chest Hospital Shanghai Jiao Tong University Shanghai China

**Keywords:** atherosclerosis, circ_0002984, miR‐326‐3p, ox‐LDL, VAMP3

## Abstract

Atherosclerosis can result in multiple cardiovascular diseases. Circular RNAs (CircRNAs) have been reported as significant non‐coding RNAs in atherosclerosis progression. Dysfunction of vascular smooth muscle cells (VSMCs) is involved in atherosclerosis. However, up to now, the effect of circ_0002984 in atherosclerosis is still unknown. Currently, we aimed to investigate the function of circ_0002984 in VSMCs incubated by oxidized low‐density lipoprotein (ox‐LDL). Firstly, our findings indicated that the expression levels of circ_0002984 were significantly up‐regulated in the serum of atherosclerosis patients and ox‐LDL‐incubated VSMCs. Loss of circ_0002984 suppressed VSMC viability, cell cycle distribution and migration capacity. Then, we carried out ELISA assay to determine TNF‐α and IL‐6 levels. The data implied that lack of circ_0002984 obviously repressed ox‐LDL–stimulated VSMC inflammation. Meanwhile, miR‐326‐3p, which was predicted as a target of circ_0002984, was obviously down‐regulated in VSMCs treated by ox‐LDL. Additionally, after overexpression circ_0002984 in VSMCs, a decrease in miR‐326‐3p was observed. Subsequently, miR‐326‐3p was demonstrated to target vesicle‐associated membrane protein 3 (VAMP3). Therefore, we hypothesized that circ_0002984 could modulate expression of VAMP3 through sponging miR‐326‐3p. Furthermore, we confirmed that up‐regulation of miR‐326‐3p rescued the circ_0002984 overexpressing‐mediated effects on VMSC viability, migration and inflammation. Additionally, miR‐326‐3p inhibitor‐mediated functions on VSMCs were reversed by knockdown of VAMP3. In conclusion, circ_0002984 mediated cell proliferation, migration and inflammation through modulating miR‐326‐3p and VAMP3 in VSMCs, which suggested that circ_0002984 might hold great promise as a therapeutic strategy for atherosclerosis.

## 1 INTRODUCTION

Atherosclerosis is a common cause of various vascular diseases.^1^ The disease resulted from atherosclerosis is becoming a serious concern. Atherosclerosis can induce formation of thrombus through disrupting the integrity of arterial surface.^2^ It is significant to identify the possible mechanism of atherosclerosis development. Its common lesions of atherosclerosis can include sclerosis, stenosis and atherosclerotic plaque formation.^3^, ^4^ Recently, VSMCs are reported to participate in the remodelling of arterial wall.^5^ The viability and migration of VSMCs act a crucial role in atherosclerosis progression.^6^, ^7^ Therefore, it is significant to find out the detailed mechanisms of atherosclerosis.

CircRNAs contain a covalently closed continuous loop, and they are single‑stranded RNAs.^8^ CircRNAs are resistant to the degradation mediated by exonuclease.^9^ Many studies report that there is a close link between atherosclerosis progression and circRNAs.^10^, ^11^ For instance, circ‐Sirt1 can control NF‐κB through enhancing SIRT1 via binding to miR‐132/212 in VSMCs.^12^ In addition, circ_0003204 can repress endothelial cell proliferation and migration through sponging miR‐370‐3p in atherosclerosis progression.^13^ Previously, microarray analysis has indicated that circ_0002984 is elevated in VSMCs treated with ox‐LDL.^14^ The mechanism of circ_0002984 in atherosclerosis progression needs more investigation.

MicroRNAs can represent a kind of endogenous non‐coding RNAs with approximately 19‐22 nucleotides.^15^ Through complementarily binding their seed sequences with their targeting mRNA, microRNAs transcriptionally inhibit it to modulate post‐transcriptional gene expression.^16^ MicroRNAs can exhibit crucial roles in regulating atherosclerosis.^17^, ^18^ Recently, it has been reported that the loss of miR‐326‐3p participates in various diseases. However, whether miR‐326‐3p may exhibit a regulatory role in VSMCs remains poorly known.

Based on these backgrounds, our research aimed to explore the biological function of circ_0002984 in atherosclerosis. We hypothesize that circ_0002984 regulates VAMP3 expression mainly through miR‐326‐3p to promote atherosclerosis progression. In our study, we implied that circ_0002984 was increased in atherosclerosis, which resulted in the down‐regulation of miR‐326‐3p in VSMCs, consequently leading to the enhancement of VAMP3 and the progression of AS.

## 2 MATERIALS AND METHODS

### 2.1 Clinical samples

Our study was done based on the approval of Medical Ethical Committee in Shanghai Chest Hospital, Shanghai Jiao Tong University. Blood samples from atherosclerosis patients (n = 30) and healthy controls (n = 30) were collected. If the patient had other clinical diseases, the patient was excluded. Madison ultrasound system (Jeju, South Korea) with transducer frequency of 7.5 MHz was utilized to evaluate plaque site and range as well as internal and middle membrane thickness. Meanwhile, two neurologists with over 10‐year clinical experience segmented the plaques in ultrasound videos of the common carotid artery. The significant differences for age, sex, smoking and drinking cases were analysed between the atherosclerotic group and the control group and shown in Table 1. Serums were extracted through 1000 *g* centrifugation at room temperature. Informed consents from participants were obtained before enrolment.

**TABLE 1:** Clinical characteristics between atherosclerotic group and control group

Parameters	Atherosclerotic group (n = 30)	Control group (n = 30)	*P* values
Age	57.90 ± 11.32	56.03 ± 9.46	0.336
Sex, male cases	19	16	0.780
Smoking, cases	13	14	0.490
Drinking, cases	8	7	0.643
Hypertension, cases	23	5	0.000
Diabetes, cases	16	8	0.002

### 2.2 Cell lines

VSMCs were purchased from ATCC (Manassas, VA, USA). Cells were incubated in McCoy's 5A medium (Sigma) supplemented with 10% foetal bovine serum (FBS, Sigma) in 5% CO_2_ at 37°C. ox‐LDL (Sigma) was utilized to make an aberrant lipid environment.

### 2.3 Cell transfection

Circ_0002984 OE plasmids (1 µg/mL), circ_0002984 siRNA (50 nmol), miR‐326‐3p mimics (50 nmol), inhibitors (100 nmol), VAMP3 siRNA (50 nmol) were constructed by GeneChem. Cells were grown into 6‐well plates and when the cell reached 70% confluency, the transfection was conducted in VSMCs using Lipofectamine™ 3000. After 6 hours, the medium was changed using normal medium.

### 2.4 Cell Counting Kit‐8 (CCK‐8) assay

VSMCs were seeded into 96‐well plates (3 × 10^3^ per well) overnight. Cell viability was tested using a CCK‐8 assay (Beyotime). In brief, 10 µL CCK‐8 solution was added to the wells for 2 hours at 37°C. Subsequently, the absorbance of VSMCs at 450 nm was assessed under a microplate reader (Thermo Fisher Scientific).

### 2.5 Cell cycle analysis

Cells were fixed using 1 mL cold 70% ethanol for a whole night at −20°C before measuring. Then, the centrifugation of suspension was carried out at 1000 *g* for 5 minutes and we removed the ethanol. Cells were washed using PBS, and afterwards, PI solution in the presence of 1% RNase A was added. After incubated with no light at 37°C, cell cycle analysis was tested using flow cytometry.

### 2.6 Transwell assay

Transwell chambers (Beijing Solarbio) were employed to assess cell migratory capacity. Cells were grown on the upper chamber. Then, the medium with 10% FBS was loaded in the lower chamber. After crystal violet was added for 10 minutes, the cells were counted using an inverted light microscope (Olympus). Five visual fields were randomly selected to calculate the number of cells. All experiments were carried out 3 times, and the results were exhibited using average values.

### 2.7 qRT‐PCR

Total RNA from clinical samples and cells was extracted by TRIzol reagent and reverse‐transcribed to cDNA using a PrimeScript RT Master Mix kit (Takara Biotechnology). The qPCR was performed using a SYBR^®^ Green PCR Master Mix (Vazyme Biotech). PCR was carried out in a reaction volume of 10 µL, with 5 µL 2× PCR master mix (SYBR Premix Ex Taq), 0.5 µL of PCR Primer, 2 µL of cDNA and diluted to 20 µL ddH_2_O. The quantitative real‐time reaction was set at an initial denaturation step of 3 minutes at 94℃; and 94℃ 10s, 58℃ 40s, 94℃ 10s in 45 cycles, with a step from 58 to 94℃. Data were analysed using $2^{‐\Delta \Delta C_{t}}$ method. Meanwhile, circ_0002984, VAMP3, IL‐6 and TNF‐α expression was normalized to glyceraldehyde‐3‐phosphate dehydrogenase (GAPDH). U6 was utilized as the control of miR‐326‐3p. Primers were displayed in Table S1.

### 2.8 Western blot

Proteins were isolated by radio immunoprecipitation (RIPA) buffer. Protein concentration was tested using Bradford Protein Quantification Kit (Vazyme Biotech). Then, equal protein was separated by 10% sodium dodecyl sulphate‐polyacrylamide gel electrophoresis (SDS‐PAGE) and subsequently transferred to polyvinylidene fluoride (PVDF) membrane. Afterwards, the membranes were blocked with 5% skimmed milk for 1 hour. The membranes were incubated with the primary antibodies: anti‐VAMP3 and GAPDH (Abcam) at 4°C. Next day, the membranes were incubated using secondary antibody for 2 hours. Subsequently, the membranes were analysed using an electrochemiluminescence (ECL) kit (Vazyme Biotech) on ChemiDoc™ MP Imaging System.

### 2.9 Enzyme‐linked immunosorbent (ELISA) assay

The supernatants of VSMCs were collected, and concentrations of cytokines (TNF‐α and IL‐6) were evaluated by ELISA kits (R&D Systems).

### 2.10 Dual‐luciferase reporter assay

WT sequences of circ_0002984 or VAMP3 3’UTR with the target sites of miR‐370‐3p were inserted into pGL3 vectors (Promega). Luciferase reporter vectors circ_0002984 WT or VAMP3 3'UTR WT were generated. circ_0002984 MUT and VAMP3 3'UTR MUT reporter vectors were established through mutating miR‐326‐3p. The vectors were co‐transfected with miR‐326‐3p mimics or inhibitors by Lipofectamine^®^ 3000. Dual‐Glo Luciferase Assay System kit (Promega) was carried out to assess the luciferase activity.

### 2.11 RNA immunoprecipitation (RIP) assay

RIP was conducted via carrying out a Magna RIP RNA‐Binding Protein Immunoprecipitation kit. VSMCs were lysed with 200 µL RIP buffer. Magnetic beads conjugated with an anti‐Ago2 antibody or anti‐IgG antibody were used. RT‐qPCR analysis was utilized to analyse immunoprecipitated RNA.

### 2.12 Statistical analysis

Data were analysed by GraphPad Prism 7. Student's *t* test was utilized to analyse the differences between two independent groups. Then, one‐way analysis of variance was carried out to assess the differences among multiple groups. Tukey's post hoc test was carried out following ANOVA *P* < 0.05 was considered to demonstrate statistical significance.

## 3 RESULTS

### 3.1 Circ_0002984 expression was elevated in VSMCs induced by ox‐LDL

**FIGURE 1:**
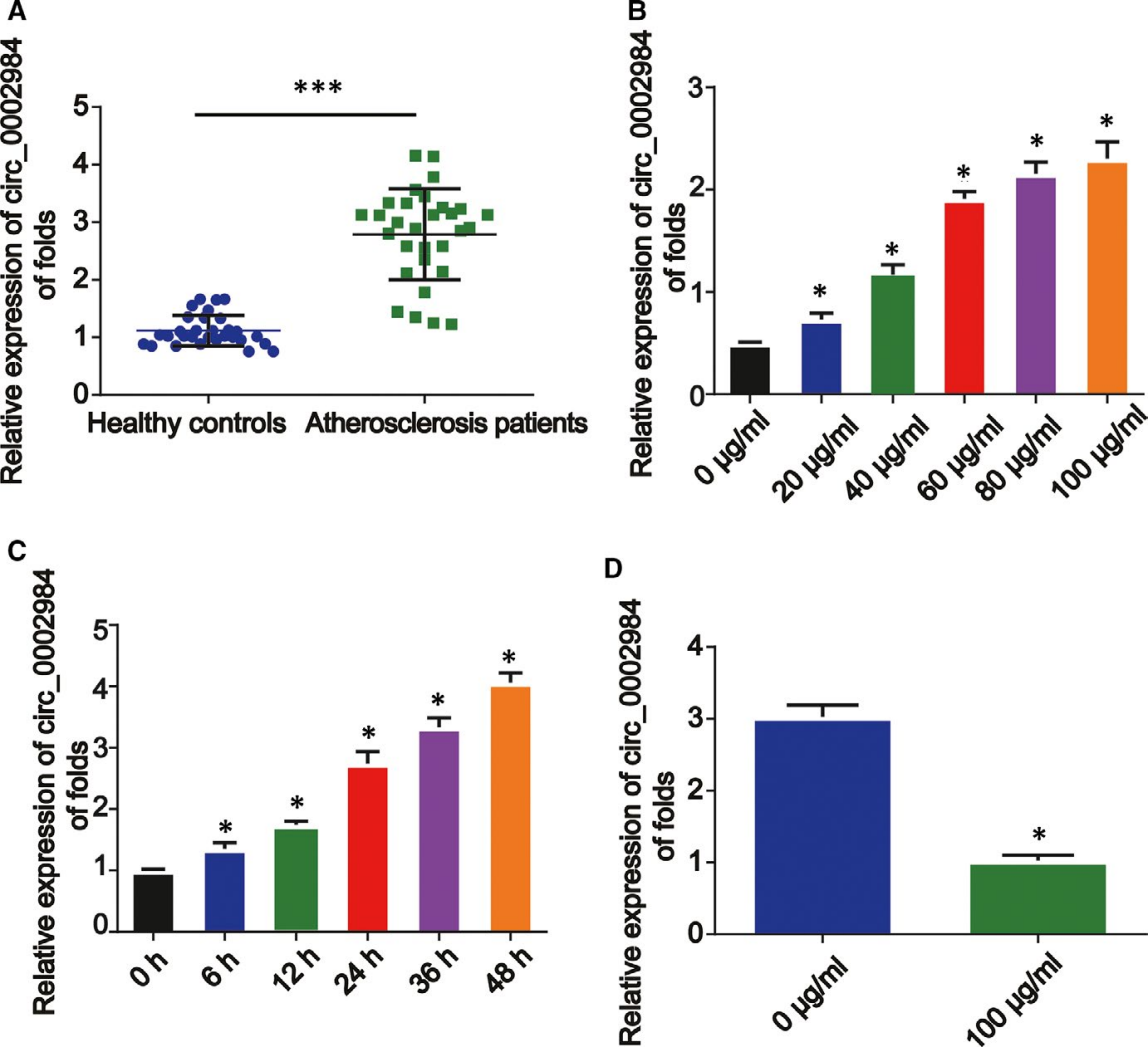
Circ_0002984 expression in VSMCs exposed to ox‐LDL. A, Relative expression of circ_0002984 in the serum of atherosclerosis patients (n = 30) and healthy controls (n = 30). B, VSMCs were incubated with 20‐100 μg/mL of ox‐LDL. C, VSMCs were treated with ox‐LDL for 0, 6, 12, 24, 36 or 48 h. D, Expression of circ_0002984 secreted in culture medium in the VSMC incubated with 100 mg/L ox‐LDL for 24 h. Compared with the control group, *P < 0.05; **compared with healthy group, *P < 0.001

Firstly, we detected circ_0002984 expression in atherosclerosis patients. As shown in Figure 1A, circ_0002984 was significantly increased in atherosclerosis serum (*P* < 0.001). To study whether circ_0002984 exhibited a role in VSMCs, we assessed whether circ_0002984 level was modulated by ox‐LDL. Firstly, VSMCs were incubated with ox‐LDL at various concentrations (0, 20, 40, 60, 80 or 100 mg/L) to mimic atherosclerosis in vitro. We observed that circ_0002984 was increased by ox‐LDL exposure (Figure 1B, *P* < 0.05). Then, 100 mg/L was used to treat VSMCs and circ_0002984 was tested at different time points. A time‐dependent enhancement of circ_0002984 was displayed in response to ox‐LDL (Figure 1C, *P* < 0.05). In Figure 1D, we have shown an up‐regulation of circ_0002984 secreted in culture medium in the VSMC incubated with 100 mg/L ox‐LDL for 24 hours (*P* < 0.05). In conclusion, circ_0002984 was increased in atherosclerosis, and herein, it was important to investigate the effect of circ_0002984.

### 3.2 Circ_0002984 increases the proliferation and migration of VSMCs

**FIGURE 2:**
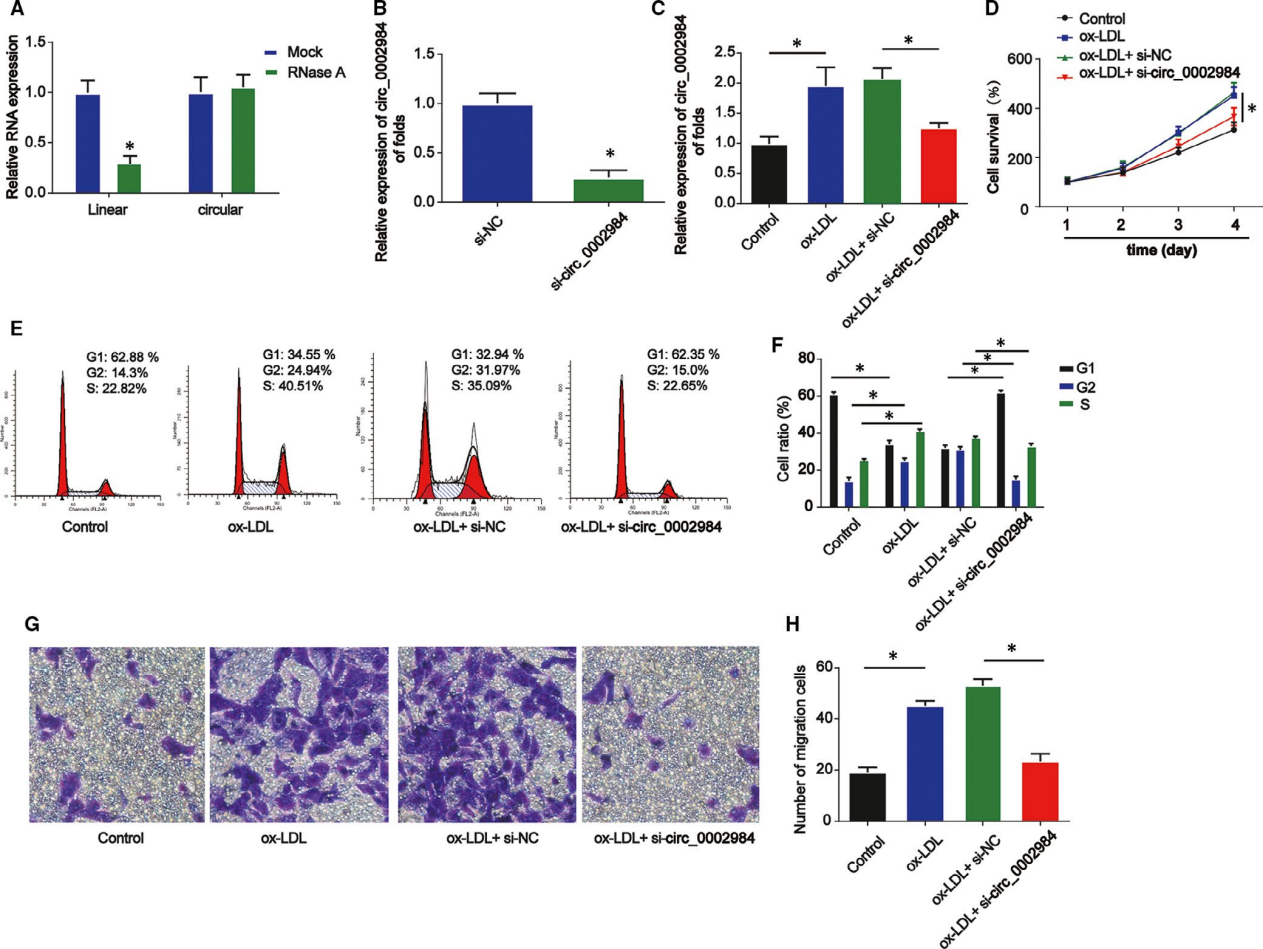
Circ_0002984 enhances the viability and migration of VSMCs indicated with ox‐LDL. A, Expression level of circ_0002984 in VSMCs treated with RNase R. B, The level of circ_0002984 in VSMCs. Cells were transfected with si‐circ_0002984 or si‐NC for 24 h. C, The level of circ_0002984 in VSMCs. Cells were treated with 100 µg/L ox‐LDL or ox‐LDL +si‐circ_0002984 for 24 h. D, The viability of VSMCs was detected by CCK‐8. E, F, The cell cycle distribution was detected by flow cytometry assay in VSMCs. G, H, The migration of VSMCs was evaluated using Transwell assay. Five visual fields were randomly selected to calculate the number of cells. Compared with the control group, *P < 0.05

Next, the function of circ_0002984 in ox‐LDL‐loaded VSMCs was focused on. We demonstrated that circ_0002984 was more resistant to RNase R in comparison with linear UBR4 mRNA (Figure 2A, *P* < 0.05). In Figure 2B, circ_0002984 expression was significantly reduced by circ_0002984 siRNA in VSMCs (*P* < 0.05). VSMCs were indicated with ox‐LDL (100 mg/L), ox‐LDL +si‐NC or ox‐LDL +si‐circ_0002984 for 24 hours. Efficiency of si‐circ_0002984 was verified using RT‐qPCR (Figure 2B, *P* < 0.05). CCK‐8 assay evidenced that down‐regulation of circ_0002984 strongly repressed viability in VSMCs as shown in Figure 2C (*P* < 0.05). Additionally, loss of circ_0002984 decreased cell cycle distribution triggered by ox‐LDL (Figure 2D,E, *P* < 0.05). The migration of VSMCs was inhibited by circ_0002984 knockdown (Figure 2F,G, *P* < 0.05). These implied circ_0002984 could act a significant role in ox‐LDL‐induced VSMC viability and migration.

### 3.3 Circ_0002984 activates IL‐6 and TNF‐α releases following ox‐LDL treatment

**FIGURE 3:**
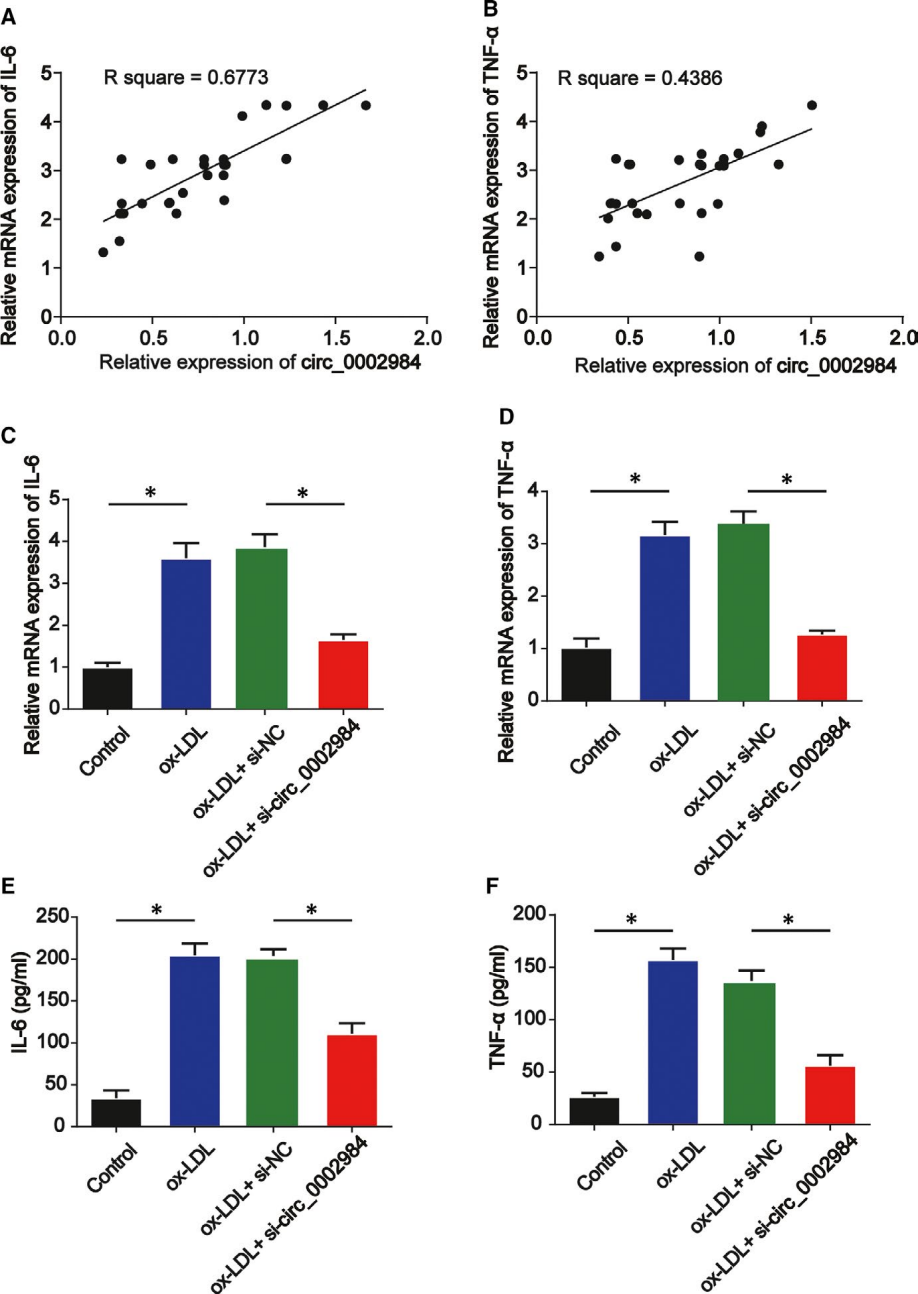
Circ_0002984 induces IL‐6 and TNF‐α production following ox‐LDL stimulation. A, Correlation between mRNA expression of IL‐6 and circ_0002984 in human patients. B, Correlation between mRNA expression of TNF‐α and circ_0002984 in human patients. C, D, IL‐6 and TNF‐α mRNA levels were determined by RT‐qPCR. E, F, Concentration of IL‐6 and TNF‐α in VSMCs was tested using ELISA assay. Compared with the control group, *P < 0.05

Inflammatory process plays a key role in atherosclerosis. We observed a positive correlation between IL‐6 and circ_0002984 as shown in Figure 3A (*P* < 0.05). A positive correlation between TNF‐α and circ_0002984 was also indicated in Figure 3B. Then, RT‐qPCR and ELISA were used to evaluate the pro‐inflammatory cytokines including IL‐6 and TNF‐α in VSMCs. In Figure 3C,D (*P* < 0.05), IL‐6 and TNF‐α mRNA levels were reduced by loss of circ_0002984. In addition, in Figure 3E,F, concentration of IL‐6 and TNF‐α in VSMCs increased by ox‐LDL was significantly reversed after circ_0002984 siRNA transfection (*P* < 0.05).

### 3.4 Circ_0002984 acted as a sponge of miR‐326‐3p in ox‐LDL‐induced VSMCs

**FIGURE 4:**
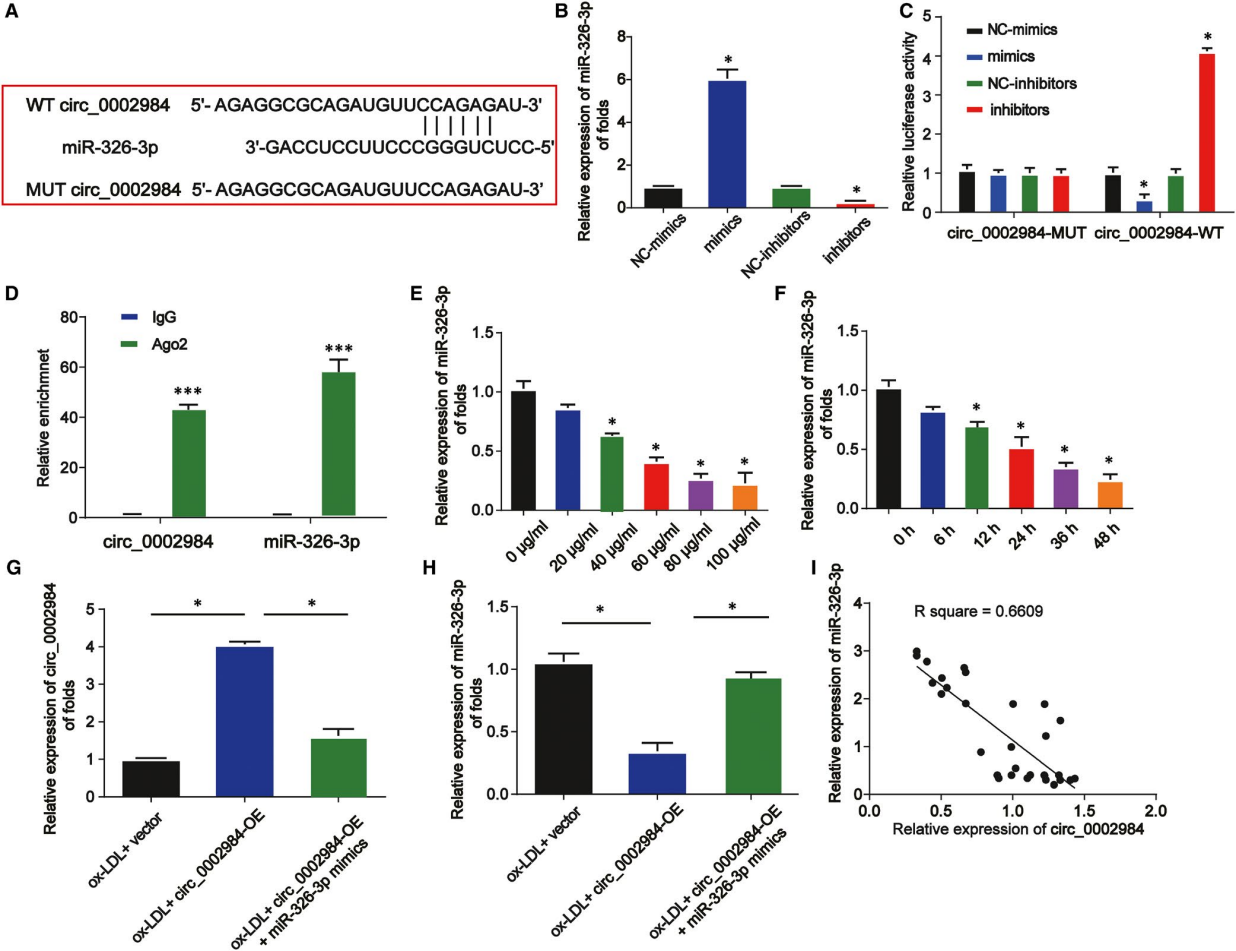
Circ_0002984 acts as a sponge of miR‐326‐3p. A, The putative binding sites between circ_0002984 and miR‐326‐3p. B, miR‐326‐3p expression in VSMCs transfected with miR‐326‐3p mimics or inhibitors. C, The luciferase activity of circ_0002984 WT and circ_0002984 MUT was detected in VSMCs. D, RIP assay was conducted to determine the interaction between circ_0002984 and miR‐326‐3p. E, The expression of miR‐326‐3p in VSMCs treated with various doses of ox‐LDL for 24 h was assessed. F, The expression of miR‐326‐3p in VSMCs treated with 100 µg/mL ox‐LDL for various times. G, Expression of circ_0002984 in VSMCs. Cells were treated with 100 µg/mL ox‐LDL, ox‐LDL +circ_0002984‐OE or ox‐LDL +circ_0002984‐OE +miR‐326‐3p mimics. H, The expression of miR‐326‐3p in VSMCs. I, Correlation between miR‐326‐3p and circ_0002984 in human patients. Compared with the control group, *P < 0.05; compared with the IgG group, *P < 0.001

To further understand the regulatory relationship between and miRNAs, the miRNA targets for circ_0002984 were predicted in our study by starBase database. In Figure 4A, miR‐326‐3p was predicted to have the binding sites of circ_0002984. In Figure 4B, miR‐326‐3p was increased by miR‐326‐3p mimics while decreased by miR‐326‐3p inhibitors (*P* < 0.05). Dual‐luciferase and RIP assays were carried out. It was revealed miR‐326‐3p significantly reduced the luciferase activity of circ_0002984 WT (Figure 4C, *P* < 0.05). Additionally, RIP assay proved the enrichment of circ_0002984 and miR‐326‐3p was increased in anti‐Ago2 group (Figure 4D, *P* < 0.001). In Figure 4E,F, the expression of miR‐326‐3p was decreased in VSMCs by ox‐LDL in a dose‐ and time‐dependent course (*P* < 0.05). Then, in Figure 4G,H, circ_0002984 overexpression decreased miR‐326‐3p level in VSMCs (*P* < 0.05). In addition, a negative correlation between miR‐326‐3p and circ_0002984 was exhibited in Figure 4I. These indicated circ_0002984 could sponge miR‐326‐3p (*P* < 0.05).

### 3.5 Circ_0002984 modulates cell viability, migration and inflammation through targeting miR‐326‐3p in VSMCs incubated with ox‐LDL

**FIGURE 5:**
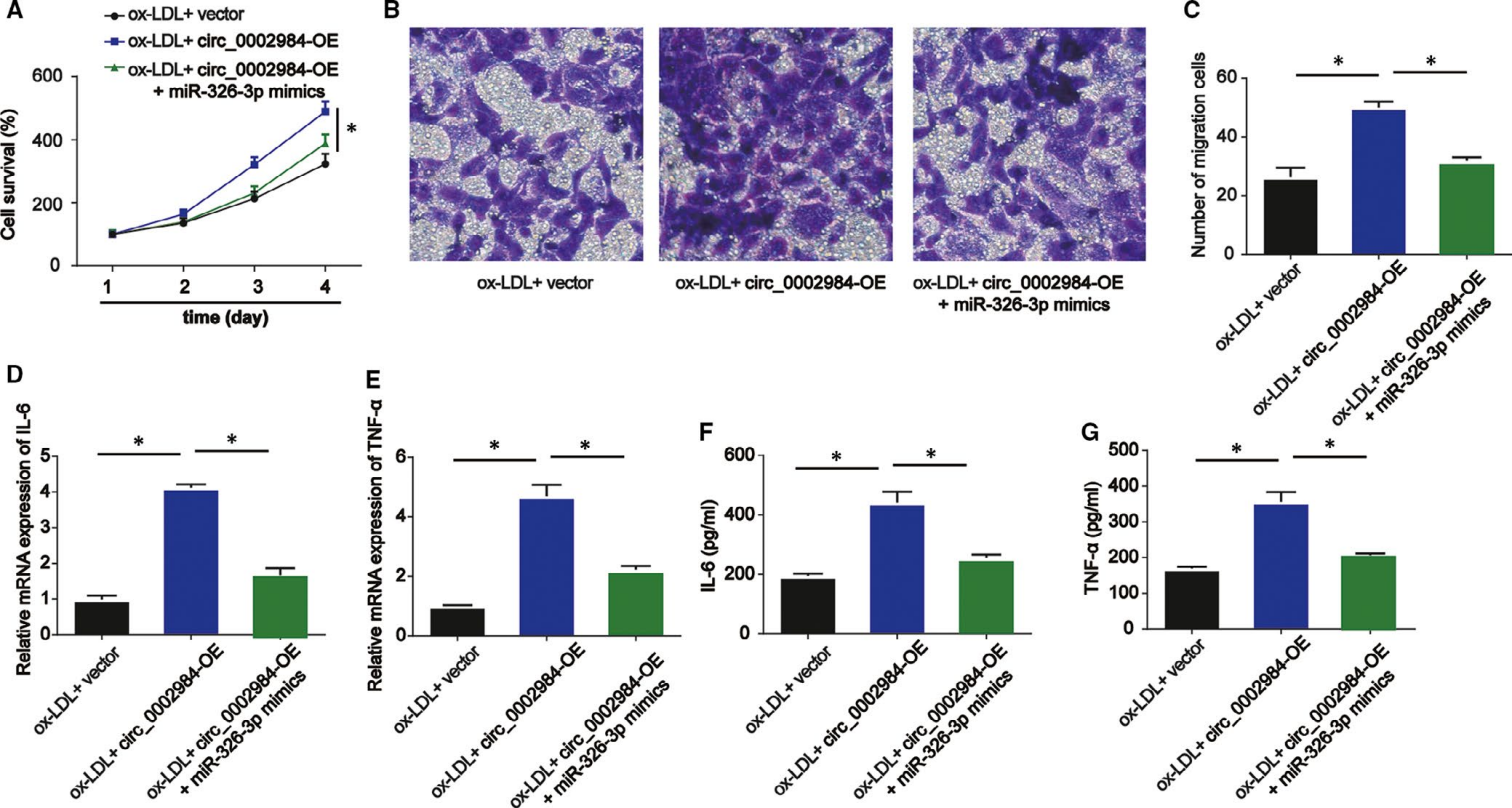
Circ_0002984 regulates cell viability, migration and inflammation via targeting miR‐326‐3p in VSMCs incubated with ox‐LDL. A, The viability of VSMCs treated with 100 µg/mL ox‐LDL, ox‐LDL +circ_0002984‐OE or ox‐LDL +circ_0002984‐OE +miR‐326‐3p mimics was tested by CCK‐8 assay. B, C, The migration of VSMCs was evaluated by Transwell assay. Five visual fields were randomly selected to calculate the number of cells. D, E, IL‐6 and TNF‐α mRNA levels were determined by RT‐qPCR. F, G, Concentration of IL‐6 and TNF‐α was tested by ELISA assay. Compared with the control group, *P < 0.05

Moreover, VSMCs were incubated with 100 µg/mL ox‐LDL, ox‐LDL +circ_0002984‐OE or ox‐LDL +circ_0002984‐OE +miR‐326‐3p mimics for 24 hours. CCK‐8 assay was carried out and miR‐326‐3p reduced the circ_0002984‐OE–induced viability of ox‐LDL‐indicated VSMCs (*P* < 0.05) as shown in Figure 5A. The circ_0002984‐OE–mediated roles on cell migration were rescued by miR‐326‐3p in Figure 5B,C (*P* < 0.05). In Figure 5D–G, co‐transfection with circ_0002984‐OE +miR‐326‐3p mimics partially repressed the effects of circ_0002984‐OE on the inflammation releases (*P* < 0.05).

### 3.6 Circ_0002984 modulates VAMP3 expression by sponging miR‐326‐3p

**FIGURE 6:**
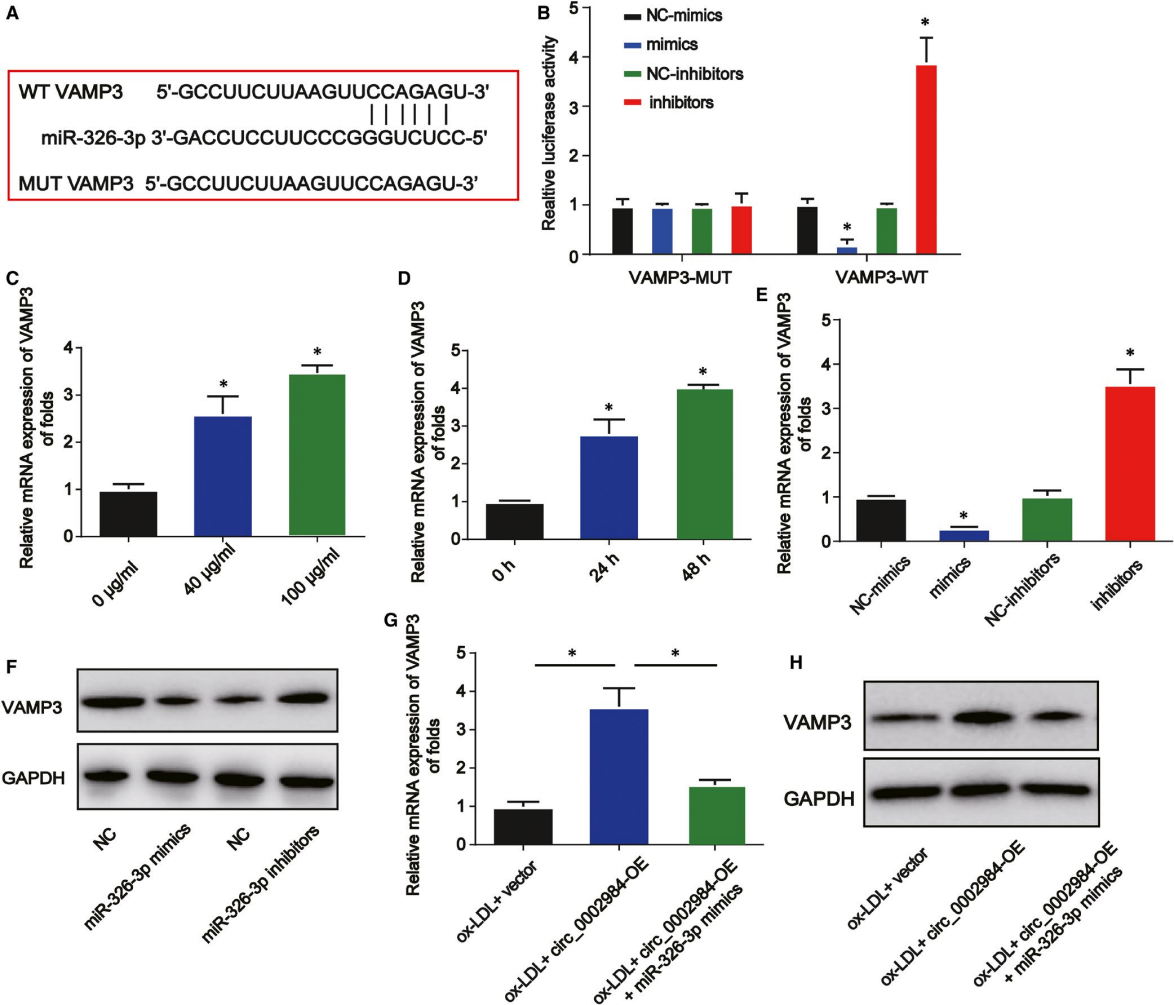
Circ_0002984 regulates VAMP3 via sponging miR‐326‐3p. A, The potential target sites between miR‐326‐3p and VAMP3. B, The luciferase activity of VAMP3 3'UTR WT and VAMP3 3'UTR MUT. C, D, VAMP3 mRNA expression in VSMCs indicated with different doses of ox‐LDL or 100 μg/mL ox‐LDL at different time. E, F, The expression of VAMP3 in VSMCs transfected with the miR‐370‐3p mimics or inhibitors. G, H, The expression of VAMP3 in VSMCs treated with 100 µg/mL ox‐LDL, ox‐LDL +circ_0002984‐OE or ox‐LDL +circ_0002984‐OE +miR‐326‐3p mimics. Compared with the control group, *P < 0.05

To determine the mechanism of miR‐326‐3p, we identified miR‑326‐3p could bind to the 3'UTR of VAMP3 using starBase database (Figure 6A). Their interaction was verified using the dual‐luciferase reporter assay (Figure 6B). VAMP3 mRNA expression was elevated in VSMCs dose dependently and time dependently (Figure 6C,D, *P* < 0.05). VAMP3 was up‐regulated by miR‑326‐3p inhibitors while reduced by miR‑326‐3p mimics in Figure 6E,F (*P* < 0.05). In addition, overexpression of circ_0002984 increased VAMP3 expression, whereas miR‐326‐3p reversed this (Figure 6G,H, *P* < 0.05). Taken together, these indicated VAMP3 was a target of miR‐326‐3p, and circ_0002984 positively modulated VAMP3 via miR‐326‐3p.

### 3.7 Lack of VAMP3 reverses the anti‐miR‐370‐3p–mediated function on VSMCs

**FIGURE 7:**
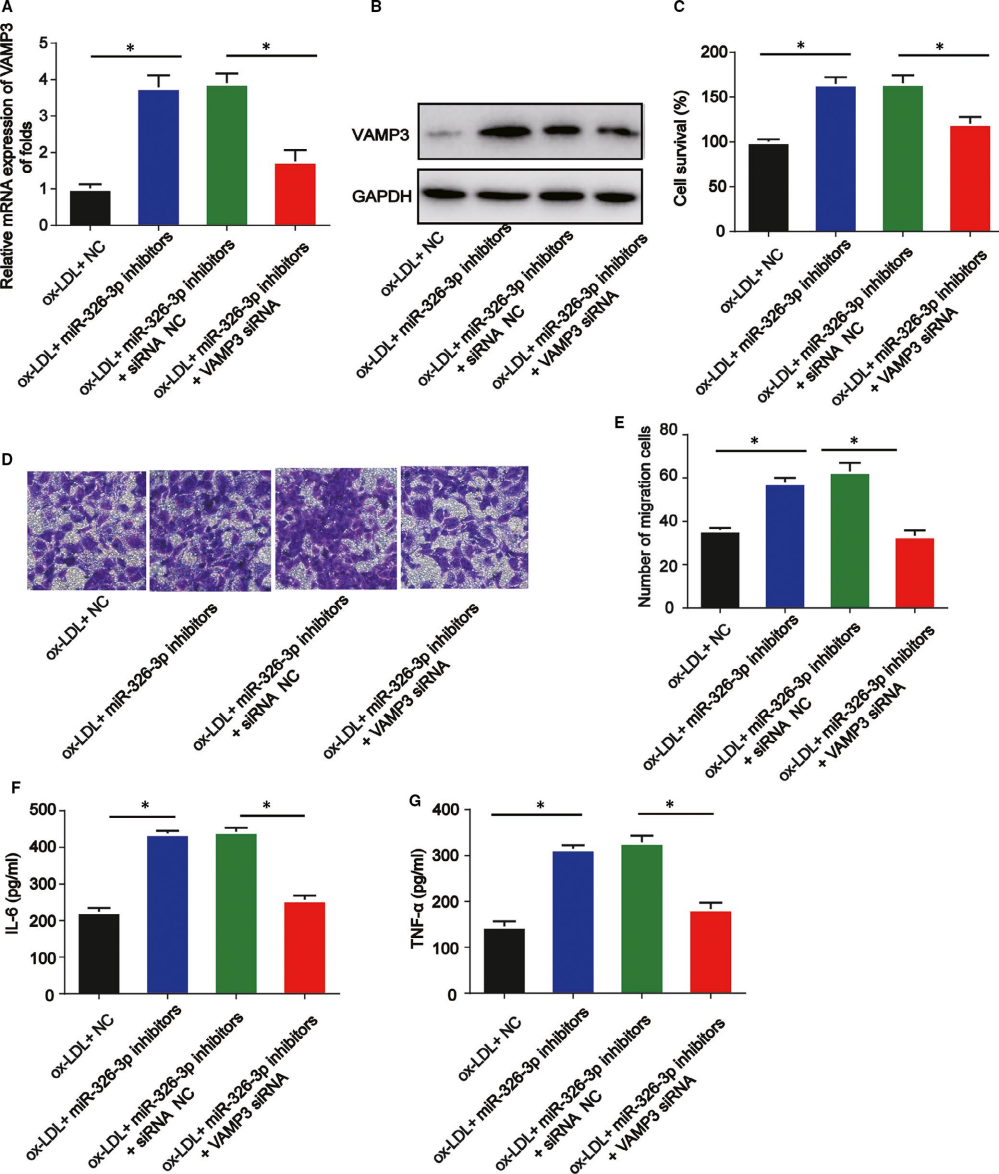
Lack of VAMP3 reverses the anti‐miR‐370‐3p–mediated effects on VSMC viability, migration and inflammation. VSMCs were treated with 100 µg/mL ox‐LDL, ox‐LDL +miR‐326‐3p inhibitors or ox‐LDL +miR‐326‐3p inhibitors +VAMP3 siRNA. A, B, The expression of VAMP3. C, Cell viability was detected by CCK‐8 assay. D, E, cell migration was evaluated by Transwell assay. Five visual fields were randomly selected to calculate the number of cells. F, G, The protein levels of IL‐6 and TNF‐α were determined using ELISA assay. Compared with the control group, *P < 0.05

Furthermore, to explore the relationship between miR‐326‐3p and VAMP3 in atherosclerosis, VSMCs were exposed to 100 µg/mL ox‐LDL, ox‐LDL +miR‐326‐3p inhibitors or ox‐LDL +miR‐326‐3p inhibitors +VAMP3 siRNA. The expression of VAMP3 was enhanced by miR‐326‐3p inhibitors while inhibited by VAMP3 siRNA in vitro (Figure 7A,B, *P* < 0.05). CCK‐8 assay indicated loss of VAMP3 obviously repressed the anti‐miR‑326‐3p–mediated increase in the cell survival of VSMCs (Figure 7C, *P* < 0.05). Anti‐miR‐326‐3p–mediated increased migration capacity was reversed by VAMP3 siRNA (Figure 7D,E, *P* < 0.05). Subsequently, as shown in Figure 7F,G, the activated inflammation induced by miR‐326‐3p inhibitors was repressed by VAMP3 silencing in VSMCs (*P* < 0.05). All these results revealed that miR‐326‐3p regulated the function of ox‐LDL‐induced VSMCs by targeting VAMP3.

## 4 DISCUSSION

It has been revealed that atherosclerosis is a serious burden to public health. Meanwhile, some reports have found that dysfunction of VSMCs is involved in atherosclerosis.^19^, ^20^ CircRNAs can manifest crucial roles in multiple human diseases. For instance, circ_0044073 is up‐regulated in atherosclerosis and it can induce VSMCs growth by targeting miR‑107.^21^ Circ_0003575 is able to regulate vascular endothelial cell proliferation induced by ox‐LDL.^22^ In addition, circ‐SATB2 modulates VSMC proliferation via miR‐939 and increase STIM1 expression.^23^

Previously, it has been reported that circ_0002984 is significantly increased in the ox‐LDL‐induced VSMCs.^14^ In our present study, we found that circ_0002984 was elevated in a concentration‐dependent and time‐dependent manner in ox‐LDL‐induced VSMCs, which was consistent with the previous study. In addition, the effects of circ_0002984 on VSMCs in response to ox‐LDL were evaluated. Knockdown of circ_0002984 could gradually repress cell viability, arrest cell cycle and inhibit cell migration dramatically. Taken these together, circ_0002984 was suggested as a diversified regulator in atherosclerosis. miR‐326‐3p was decreased in atherosclerosis, which was negatively modulated by circ_0002984. VAMP3 was predicted as a target of miR‐326‐3p. During atherosclerosis, the significance of ox‐LDL/circ_0002984/miR‐326‐3p/VAMP3 signalling in the development of atherosclerosis was established.

CircRNAs serve as microRNA sponges in human diseases.^24^ Next, we further investigate the mechanism by which circ_0002984 knockdown suppressed the progression of atherosclerosis. miR‑326‐3p was a target of circ_0002984. miR‑326‐3p is reported to be involved in diseases. For example, miR‑326‐3p can ameliorate high glucose and ox‐LDL‐IC–induced fibrotic injury in renal mesangial cells through targeting FcγRIII.^25^ miR‐326‐3p is involved in circ_0000467‐induced gastric cancer progression.^26^ In addition, miR‐326‐3p enhances cadmium‐triggered NRK‐52E cell apoptosis via down‐regulating PLD1.^27^ Here, miR‑326‐3p was down‐regulated in VSMCs treated with ox‐LDL and it was negatively modulated by circ_0002984. Next, overexpression of miR‑326‐3p reversed the circ_0002984 overexpressing‑induced effects on the viability, migration and inflammation of VSMCs. Our study firstly reported the function of miR‐326‐3p on atherosclerosis, supplementing the biological role of miR‐326‐3p in atherosclerosis.

It has been shown that microRNAs exert their biological functions which result from their target genes. VAMP3 is a SNARE protein, and it can regulate exocytosis in cell migration and integrin trafficking. For instance, loss of VAMP3 suppresses cell migration and integrin‐mediated adhesion.^28^ VAMP3 is a crucial therapeutic target for microglial activation by surgical trauma.^29^ VAMP3 is involved in endothelial weibel‐palade bodies in exocytosis.^30^ VAMP3 mediates the disturbed secretion of flow‐induced endothelial microRNAs.^31^ mRNA levels of VAMP3 were measured, and VAMP3 was up‐regulated in ox‑LDL–induced VSMCs. Additionally, VAMP3 was demonstrated to be negatively modulated by miR‑326‐3p. Circ_0002984 up‐regulated VAMP3 expression via sponging miR‑326‐3p. Loss of VAMP3 reversed the anti‐miR‑326‐3p–mediated effects on viability, migration and inflammation responses of ox‑LDL–induced VSMCs. Frankly speaking, we only focused on the effect of circ_0002984 in vitro. In our further study, the role of circ_0002984/miR‑326‐3p/VAMP3 in atherosclerosis should be proved in vivo. In addition, the mechanism of up‐regulation of circ_0002984 induced by ox‐LDL in VSMCs is unclear and needs more investigation.

To sum up, circ_0002984 could regulate the cellular development of ox‐LDL‐incubated VSMCs. We validated the function of circ_0002984 on atherosclerosis via regulating miR‐326‐3p and VAMP3. Thus, circ_0002984 might serve as a potential target for atherosclerosis treatment.

## CONFLICT OF INTEREST

The authors confirm that there are no conflicts of interest.

## AUTHOR CONTRIBUTIONS

**Ruogu Li:** Investigation (equal); Methodology (equal); Software (equal); Writing‐original draft (equal). **Qiliang Jiang:** Data curation (equal); Formal analysis (equal); Software (equal); Validation (equal). **Yue Zheng:** Conceptualization (lead); Project administration (lead); Writing‐review & editing (lead).

## Supporting information

Table S1

## Data Availability

The data that support the findings of this study are available from the corresponding author upon reasonable request.
