# Supplementary material for: Circ_0002984 induces proliferation, migration and inflammation response of VSMCs induced by ox‐LDL through miR‑326‐3p/VAMP3 axis in atherosclerosis
Source: J Cell Mol Med. 2021 Jun 25;25(16):8028–38. doi: 10.1111/jcmm.16734 (PMC8358879; doi:10.1111/jcmm.16734)
Supplement: Supplementary file 1 — Table S1 [file JCMM-25-8028-s001.docx]

**Supplementary Table 1. Primers used for real-time q-PCR**

| **Genes** | **Forward (5’-3’)** | **Reverse (5’-3’)** |
| --- | --- | --- |
| **GAPDH** | TGGGTGTGAACCATGAGAAGT | TGAGTCCTTCCACGATACCAA |
| **U6** | CTCGCTTCGGCAGCACA | AACGCTTCACGAATTTGCGT |
| **circ_****0002984** | CGGCAGCATACAGCTTTCAC | GCTGTACTGCCACACGTCTT |
| **miR-326** | CATCTGTCTGTTGGGCTGGA | AGGAAGGGCCCAGAGGCG |
| **VAMP3**  **IL-6**  **TNF-α** | ACCTCACAACTTTGGTGCTG  ACTCACCTCTTCAGAACGAATTG  CCTCTCTCTAATCAGCCCTCTG | CATTCCCAGCTAAATGCACA  CCATCTTTGGAAGGTTCAGGTTG  GAGGACCTGGGAGTAGATGAG |
